# Supplementary material for: Accurate and fast feature selection workflow for high-dimensional omics data
Source: PLoS One. 2017 Dec 20;12(12):e0189875. doi: 10.1371/journal.pone.0189875 (PMC5738110; doi:10.1371/journal.pone.0189875)
Supplement: S1 File — (DOCX) [file pone.0189875.s001.docx]

Supplementary Information

Table of Contents

[Note 1: Datasets 2](#_Toc495054613)

[Note 2: Feature Selection Approaches 3](#_Toc495054614)

[Note 3: Number of Final Features after the FS method step 5](#_Toc495054615)

[Note 4: Benchmark of the R workflow proposed with the MRMD tool 5](#_Toc495054616)

[References 6](#_Toc495054617)

# **Note 1: Datasets**

**Table 1**: Description of the genomics and proteomics datasets used for benchmarking the feature selection (FS) methods developed in this manuscript.

| # dataset | GEO accession numbers | Technology | Samples | Features | # Classes | Description | ML problem | References |
| --- | --- | --- | --- | --- | --- | --- | --- | --- |
| 1 | GSE5325 | Transcriptomics | 106 | 8534 | 2 | Analysis of breast cancer tumor samples using 2-color cDNA microarrays. | Classification | [1] |
| 2 | - | Proteomics | 7000 | 545 | - | Peptides fractionated by IPG-HPLC and analyzed by Mass spectrometry. | Regression | [2, 3] |
| 3 | - | Proteomics | 44 | 15525 | 5 | Triple-Negative Breast Cancer (TNBC) proteome. Label-free deep proteome analysis of 44 (samples and technical replicates) human breast specimens. | Classification | [4] |
| 4 | GSE48760 | Transcriptomics | 208 | 25697 | 2 | Transcriptomics analysis of left ventricles of mouse subjected to an isoproterenol challenge. | Classification | [5] |
| 5 | GSE6919  /GPL8300 | Transcriptomics | 171 | 12558 | 4 | Expression data from normal and prostate tumor tissues. | Classification | [6, 7] |
| 6 | GSE6919  /GPL92 | Transcriptomics | 168 | 12553 | 4 | Expression data from normal and prostate tumor tissues. | Classification | [6, 7] |
| 7 | GSE6919  /GPL93 | Transcriptomics | 165 | 12626 | 4 | Expression data from normal and prostate tumor tissues. | Classification | [6, 7] |

# **Note 2: Feature Selection Approaches**

**
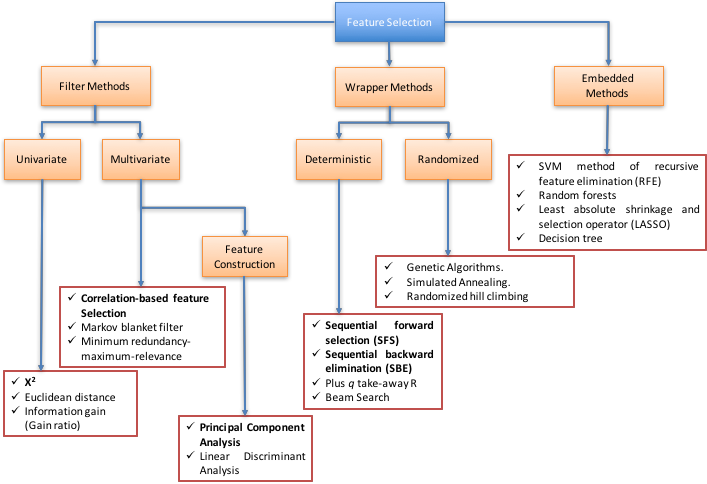
**

**Figure 1**: Schema of the existing Feature Selection Approaches. We highlighted (in **bold** letters) all the algorithms that can be combined using the proposed workflow in this study.


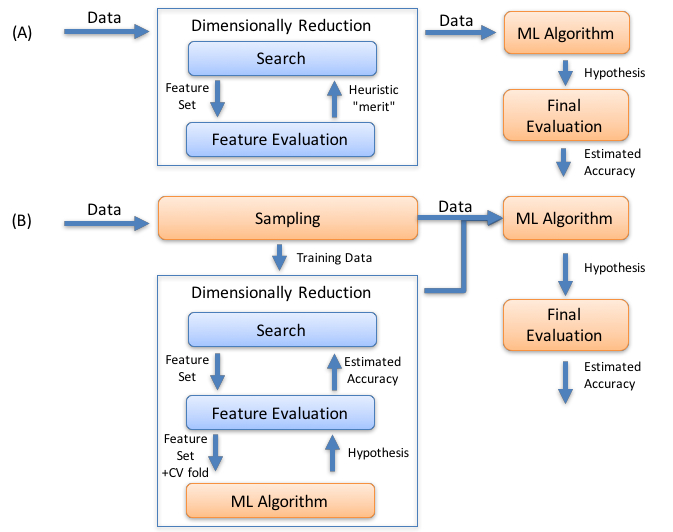


**Figure 2**: Diagram of Feature Selection (FS) Filtering (A) and (B) Wrapper approaches. In the filtering approaches the dimension reduction occurs before the machine learning step. In contrast, in the Wrapper approaches the machine learning model (ML Algorithm) is used in combination with the FS step to improve the selection process. The proposed workflow combines the strengths of both techniques.

# **Note 3: Number of Final Features after the FS method step**

**Table 2:** Number of final features after the FS step for the proposed workflow. Three different combinations are shown: The Random Forest classification algorithm without a feature selection step (**RF);** the combination of the univariate correlation filter with the matrix correlation and recursive feature elimination before the Random Forest classification step (**X2-CM-RFE-RF**); and finally, the combination of univariate correlation filter with principal component analysis, before the Random Forest classification step (**X2-PCA-RFE-RF**).

| Workflow | GSE5325  (Dataset 1) | TNBC  (Dataset 3) | GSE48760  (Dataset 4) | GSE6919  /GPL8300  (Dataset 5) | GSE6919  /GPL92  (Dataset 6) | GSE6919  /GPL93  (Dataset 7) |
| --- | --- | --- | --- | --- | --- | --- |
| RF | 1,874 | 1,419 | 4,117 | 4,803 | 4,761 | 4,941 |
| X2-CM-RFE-RF | 8 | 20 | 7 | 50 | 45 | 50 |
| X2-PCA-RFE-RF | 8 | 6 | 5 | 35 | 5 | 6 |

# **Note 4: Benchmark of the R workflow proposed with the MRMD tool**

**Table 3**. Performance evaluation on TNBC and GSE5325 datasets for MRMD tool and the proposed FS workflow(s). MRMD: Maximum-Relevance-Maximum-Distance, X2: Univariate Correlation filter, CM: Multivariate Correlation Matrix, RFE: Recursive Feature Elimination, RF: Random Forest, PCA: Principal Component Analysis, GI: Gain Information filter.

|  | TNBC | | | GSE5325 | | |
| --- | --- | --- | --- | --- | --- | --- |
|  | **Acc. Max.** | **# Features** | **Runtime (min)** | **Acc. Max.** | **# Features** | **Runtime** |
| MRMD | 0.98 | 2689 | 18.46 | 0.95 | 409 | 57.53 |
| X2-CM-RFE-RF | 1.00 | 20 | 3.03 | 0.94 | 8 | 6.39 |
| X2-PCA-RFE-RF | 0.96 | 6 | 1.99 | 0.91 | 7 | 5.26 |
| GI-CM-RFE-RF | 0.98 | 30 | 1.67 | 0.94 | 20 | 2.62 |
| GI-PCA-RFE-RF | 0.96 | 8 | 1.01 | 0.91 | 4 | 2.65 |

# **References**

1. Saal LH, Johansson P, Holm K, Gruvberger-Saal SK, She QB, Maurer M, et al. Poor prognosis in carcinoma is associated with a gene expression signature of aberrant PTEN tumor suppressor pathway activity. Proceedings of the National Academy of Sciences of the United States of America. 2007;104(18):7564-9. doi: 10.1073/pnas.0702507104. PubMed PMID: 17452630; PubMed Central PMCID: PMCPMC1855070.

2. Perez-Riverol Y, Audain E, Millan A, Ramos Y, Sanchez A, Vizcaino JA, et al. Isoelectric point optimization using peptide descriptors and support vector machines. Journal of proteomics. 2012;75(7):2269-74. doi: 10.1016/j.jprot.2012.01.029. PubMed PMID: 22326964.

3. Perez-Riverol Y, Sanchez A, Ramos Y, Schmidt A, Muller M, Betancourt L, et al. In silico analysis of accurate proteomics, complemented by selective isolation of peptides. Journal of proteomics. 2011;74(10):2071-82. doi: 10.1016/j.jprot.2011.05.034. PubMed PMID: 21658481.

4. Lawrence RT, Perez EM, Hernandez D, Miller CP, Haas KM, Irie HY, et al. The proteomic landscape of triple-negative breast cancer. Cell Rep. 2015;11(4):630-44. doi: 10.1016/j.celrep.2015.03.050. PubMed PMID: 25892236; PubMed Central PMCID: PMCPMC4425736.

5. Wang JJ, Rau C, Avetisyan R, Ren S, Romay MC, Stolin G, et al. Genetic Dissection of Cardiac Remodeling in an Isoproterenol-Induced Heart Failure Mouse Model. PLoS genetics. 2016;12(7):e1006038. doi: 10.1371/journal.pgen.1006038. PubMed PMID: 27385019; PubMed Central PMCID: PMCPMC4934852.

6. Chandran UR, Ma C, Dhir R, Bisceglia M, Lyons-Weiler M, Liang W, et al. Gene expression profiles of prostate cancer reveal involvement of multiple molecular pathways in the metastatic process. BMC Cancer. 2007;7:64. doi: 10.1186/1471-2407-7-64. PubMed PMID: 17430594; PubMed Central PMCID: PMCPMC1865555.

7. Li S, Oh S. Improving feature selection performance using pairwise pre-evaluation. BMC bioinformatics. 2016;17:312. doi: 10.1186/s12859-016-1178-3. PubMed PMID: 27544506; PubMed Central PMCID: PMCPMC4992252.
