# Supplementary material for: Accurate and fast feature selection workflow for high-dimensional omics data
Source: PLoS One. 2017 Dec 20;12(12):e0189875. doi: 10.1371/journal.pone.0189875 (PMC5738110; doi:10.1371/journal.pone.0189875)
Supplement: S2 File — (PDF) [file pone.0189875.s002.pdf]

Feature Selection (FS) workflow report

May 18, 2017

Introduction

The report summarizing the Feature Selection pipeline results.

Feature Selection workflow

Univariate canonical correlation (X2) with Multivariate Correlation filter (MC) follow by Recursive Feature Elimination (RFE) wrapped with Random Forest (RF).

Dataset

Analysis of breast cancer tumor samples using 2-color cDNA microarrays (GSE5325).

Summary stats from training phase

Table 1: Best model metrics from 10-folds cross-validation resampling.

Variables	Accuracy	Kappa	AccuracySD	KappaSD
1	0.6286	0.2505	0.138	0.2865
2	0.7143	0.4156	0.1166	0.2369
3	0.7143	0.4127	0.1506	0.3073
4	0.7286	0.4408	0.1421	0.2967
5	0.7571	0.4996	0.1355	0.281
6	0.8143	0.624	0.1176	0.2358
7	0.8	0.5907	0.1205	0.2477
8	0.8286	0.6465	0.1127	0.2315
9	0.8	0.5884	0.1205	0.2493
10	0.8286	0.642	0.09035	0.1917
15	0.8	0.5881	0.07377	0.1514
20	0.8	0.5881	0.07377	0.1514
25	0.8	0.5884	0.1205	0.243
30	0.8143	0.6188	0.1355	0.2749
35	0.8143	0.6188	0.1355	0.2749
40	0.8143	0.6188	0.1355	0.2749
45	0.8	0.5884	0.1205	0.243
50	0.8286	0.6492	0.1313	0.2668
60	0.8143	0.6188	0.1355	0.2749
70	0.8286	0.6492	0.1313	0.2668
80	0.8143	0.6188	0.1355	0.2749
90	0.8286	0.6492	0.1313	0.2668
100	0.8143	0.616	0.1355	0.2806
1178	0.8	0.5929	0.1205	0.2399

Summary stats from testing phase

Table 2: Classification metrics from twenty class-balanced and randomized runs.

run	Variables	Accuracy	Kappa	AccuracyPValue
1	1178	0.8571	0.7107	0.0003014
2	8	0.8857	0.7667	6.136e-05
3	9	0.8	0.5882	0.003999
4	7	0.8286	0.6557	0.001202
5	90	0.9429	0.8852	1.128e-06
6	5	0.8857	0.7742	6.136e-05
7	40	0.7429	0.4615	0.02786
8	60	0.8	0.595	0.003999
9	3	0.8286	0.6316	0.001202
10	15	0.9143	0.8264	9.733e-06
11	90	0.8571	0.7107	0.0003014
12	5	0.8571	0.7154	0.0003014
13	60	0.8571	0.7059	0.0003014
14	50	0.7429	0.4615	0.02786
15	2	0.8	0.595	0.003999
16	8	0.8286	0.6613	0.001202
**17**	**8**	**0.9429**	**0.8833**	**1.128e-06**
18	35	0.9143	0.8264	9.733e-06
19	100	0.8857	0.7667	6.136e-05
20	15	0.8286	0.6613	0.001202

Accuracy_Mean	Accuracy_SD	Accuracy_Max
0.85	0.05705	0.9429

Workflow runtime

6.385 minutes

Plots

Visualization of the classification using PCA

- Groups distribution on the first two Principal Components (PC1 and PC2) from the original data (without apply any FS method).

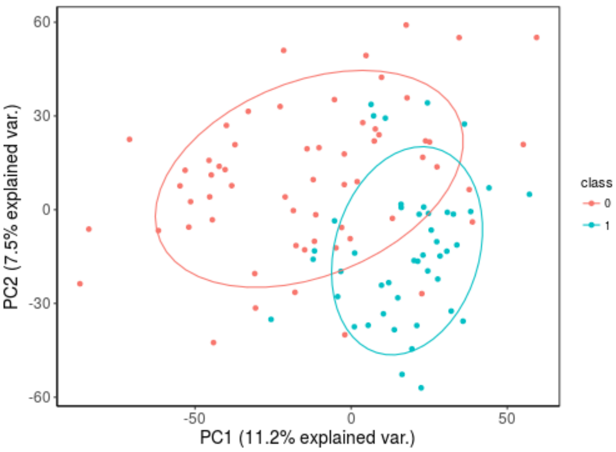

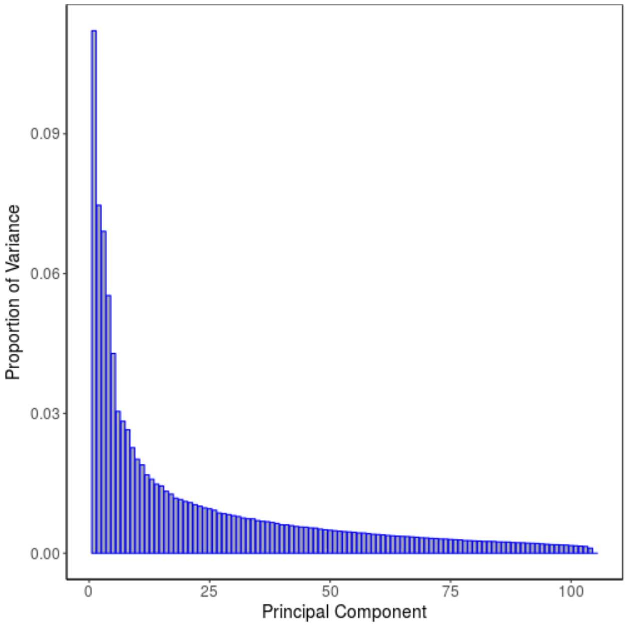

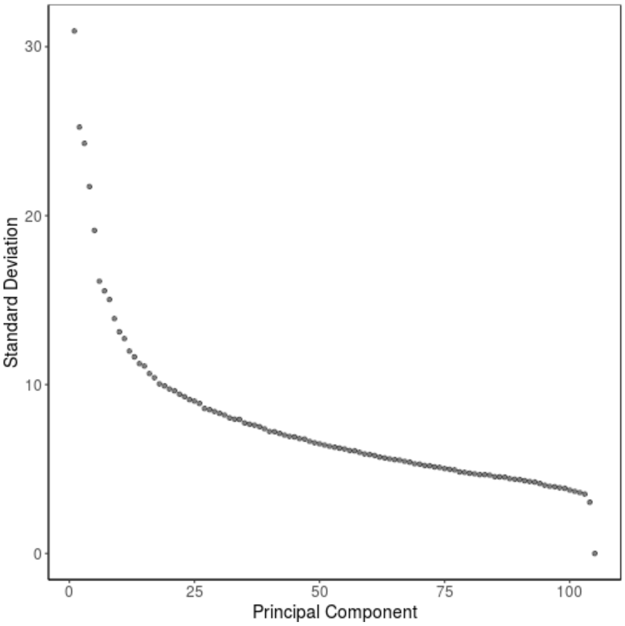

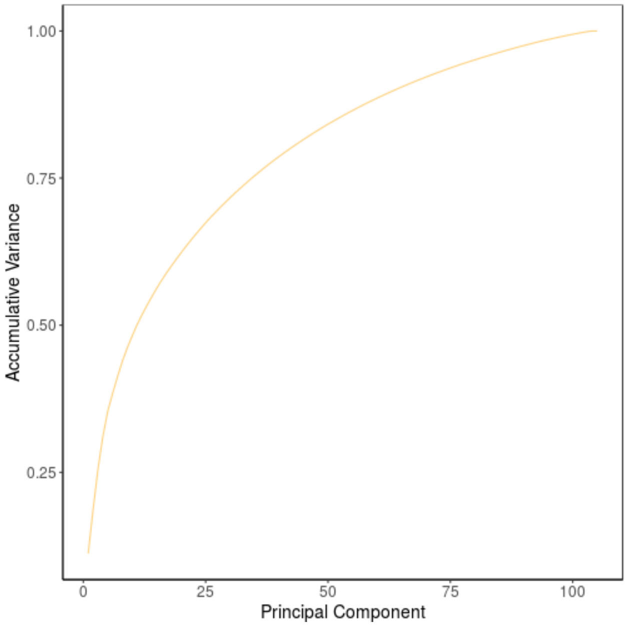

- Groups distribution on the first two Principal Components (PC1 and PC2) after to apply the FS workflow.

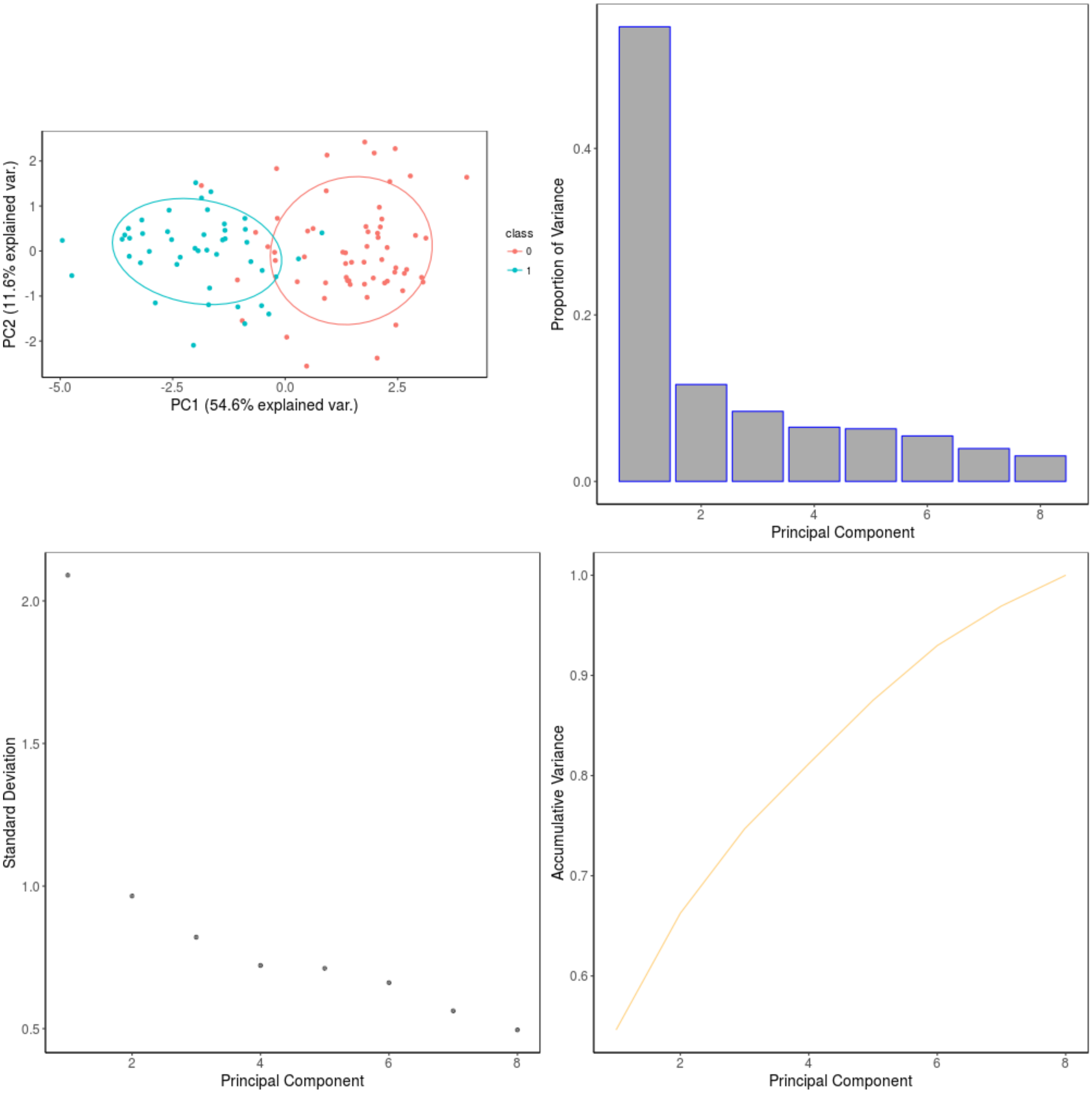

Feature Selection (FS) workflow report

May 18, 2017

Introduction

The report summarizing the Feature Selection pipeline results.

Feature Selection workflow

Univariate canonical correlation (X2) with Principal Component Analysis (PCA) follow by Recursive Feature Elimination (RFE) wrapped with Random Forest (RF).

Dataset

Analysis of breast cancer tumor samples using 2-color cDNA microarrays (GSE5325).

Summary stats from training phase

Table 1: Best model metrics from 10-folds cross-validation resampling.

Variables	Accuracy	Kappa	AccuracySD	KappaSD
1	0.8286	0.6469	0.1756	0.3633
2	0.8143	0.6263	0.1656	0.3358
3	0.8286	0.6492	0.1313	0.2724
4	0.8286	0.6446	0.1127	0.2396
5	0.7857	0.5587	0.1684	0.342
6	0.8	0.5841	0.138	0.2872
7	0.8143	0.619	0.1513	0.3123
8	0.8429	0.6796	0.1421	0.2943
9	0.8286	0.6493	0.1475	0.3051
10	0.8143	0.6145	0.1513	0.3151
15	0.8	0.5887	0.1536	0.3135
20	0.8143	0.6135	0.1911	0.4102
25	0.8286	0.6422	0.1622	0.3447
30	0.8286	0.6447	0.1313	0.2762
35	0.8286	0.6498	0.1756	0.3586
40	0.7857	0.5585	0.1388	0.2813
45	0.7857	0.5557	0.1543	0.3175
50	0.7714	0.5098	0.1807	0.3964
60	0.8	0.589	0.1677	0.3414
70	0.7714	0.5095	0.1536	0.3433
80	0.7714	0.517	0.1807	0.384
90	0.7857	0.5371	0.1543	0.3422
100	0.7571	0.4794	0.1656	0.365
105	0.7714	0.5151	0.138	0.3065

Summary stats from testing phase

Table 2: Classification metrics from twenty class-balanced and randomized runs.

run	Variables	Accuracy	Kappa	AccuracyPValue
1	3	0.8857	0.7667	6.136e-05
2	5	0.8857	0.7667	6.136e-05
3	6	0.8857	0.7705	6.136e-05
4	20	0.8286	0.6557	0.001202
5	60	0.8	0.595	0.003999
6	40	0.8857	0.7667	6.136e-05
7	15	0.7429	0.4522	0.02786
8	15	0.8857	0.7705	6.136e-05
**9**	**8**	**0.9143**	**0.8264**	**9.733e-06**
10	5	0.8	0.608	0.003999
11	8	0.8286	0.6441	0.001202
12	90	0.8286	0.6557	0.001202
13	2	0.7714	0.5254	0.01134
14	10	0.8571	0.7107	0.0003014
15	25	0.8857	0.7627	6.136e-05
16	40	0.8	0.595	0.003999
17	20	0.8857	0.7667	6.136e-05
18	10	0.8857	0.7742	6.136e-05
19	20	0.8857	0.7627	6.136e-05
20	1	0.8	0.595	0.003999

Accuracy__Mean	Accuracy__SD	Accuracy__Max
0.8471	0.04841	0.9143

Workflow runtime

5.259 minutes

Plots

Visualization of the classification using PCA

- Groups distribution on the first two Principal Components (PC1 and PC2) from the original data (without apply any FS method).

```
## PCA plot not available for this FS workflow setting
```

- Groups distribution on the first two Principal Components (PC1 and PC2) after to apply the FS workflow.

```
## PCA plot not available for this FS workflow setting
```

Feature Selection (FS) workflow report

May 18, 2017

Introduction

The report summarizing the Feature Selection pipeline results.

Feature Selection workflow

Univariate canonical correlation (X2) with Multivariate Correlation filter (MC) follow by Recursive Feature Elimination (RFE) wrapped with Random Forest (RF).

Dataset

Triple-Negative Breast Cancer (TNBC) proteome. Label-free deep proteome analysis of 44 (samples and technical repliques) human breast specimens.

Summary stats from training phase

Table 1: Best model metrics from 10-folds cross-validation resampling.

Variables	Accuracy	Kappa	AccuracySD	KappaSD
1	0.4083	0.3029	0.4129	0.4471
2	0.525	0.3418	0.3094	0.4151
3	0.5333	0.3579	0.3562	0.4732
4	0.575	0.4007	0.3631	0.4965
5	0.7083	0.62	0.2998	0.3846
6	0.7667	0.6956	0.2772	0.3638
7	0.7417	0.6623	0.3129	0.4127
8	0.7667	0.6885	0.2772	0.3672
9	0.7417	0.65	0.3129	0.4191
10	0.7917	0.7218	0.2613	0.3459
15	0.8583	0.8123	0.1927	0.2519
20	0.8917	0.8551	0.1845	0.2427
25	0.8833	0.843	0.1532	0.209
30	0.825	0.774	0.2203	0.2837
35	0.85	0.8073	0.225	0.2892
40	0.8583	0.8071	0.1524	0.2095
45	0.825	0.7766	0.2498	0.3178
50	0.8	0.7476	0.2428	0.3037
60	0.8833	0.85	0.1933	0.2439
70	0.825	0.7667	0.2498	0.3459
80	0.7833	0.6762	0.2582	0.4083
90	0.8667	0.7859	0.1851	0.3329
100	0.825	0.7667	0.2498	0.3459
757	0.8333	0.743	0.1884	0.3298

Summary stats from testing phase

Table 2: Classification metrics from twenty class-balanced and randomized runs.

run	Variables	Accuracy	Kappa	AccuracyPValue
1	50	1	1	2.216e-07
2	20	0.7692	0.7	0.000816
**3**	**20**	**1**	**1**	**2.216e-07**
4	80	1	1	2.216e-07
5	80	1	1	2.216e-07
6	70	0.8462	0.7969	9.42e-05
7	30	0.9231	0.9	6.703e-06
8	757	0.8462	0.7969	9.42e-05
9	30	0.9231	0.8992	6.703e-06
10	757	0.8462	0.803	9.42e-05
11	35	1	1	2.216e-07
12	50	1	1	2.216e-07
13	80	0.9231	0.9	6.703e-06
14	757	0.9231	0.9008	6.703e-06
15	40	0.6923	0.5938	0.004876
16	100	1	1	2.216e-07
17	45	1	1	2.216e-07
18	15	0.8462	0.8	9.42e-05
19	100	0.8462	0.803	9.42e-05
20	25	1	1	2.216e-07

Accuracy_Mean	Accuracy_SD	Accuracy_Max
0.9192	0.09161	1

Workflow runtime

3.024 minutes

Plots

Visualization of the classification using PCA

- Groups distribution on the first two Principal Components (PC1 and PC2) from the original data (without apply any FS method).

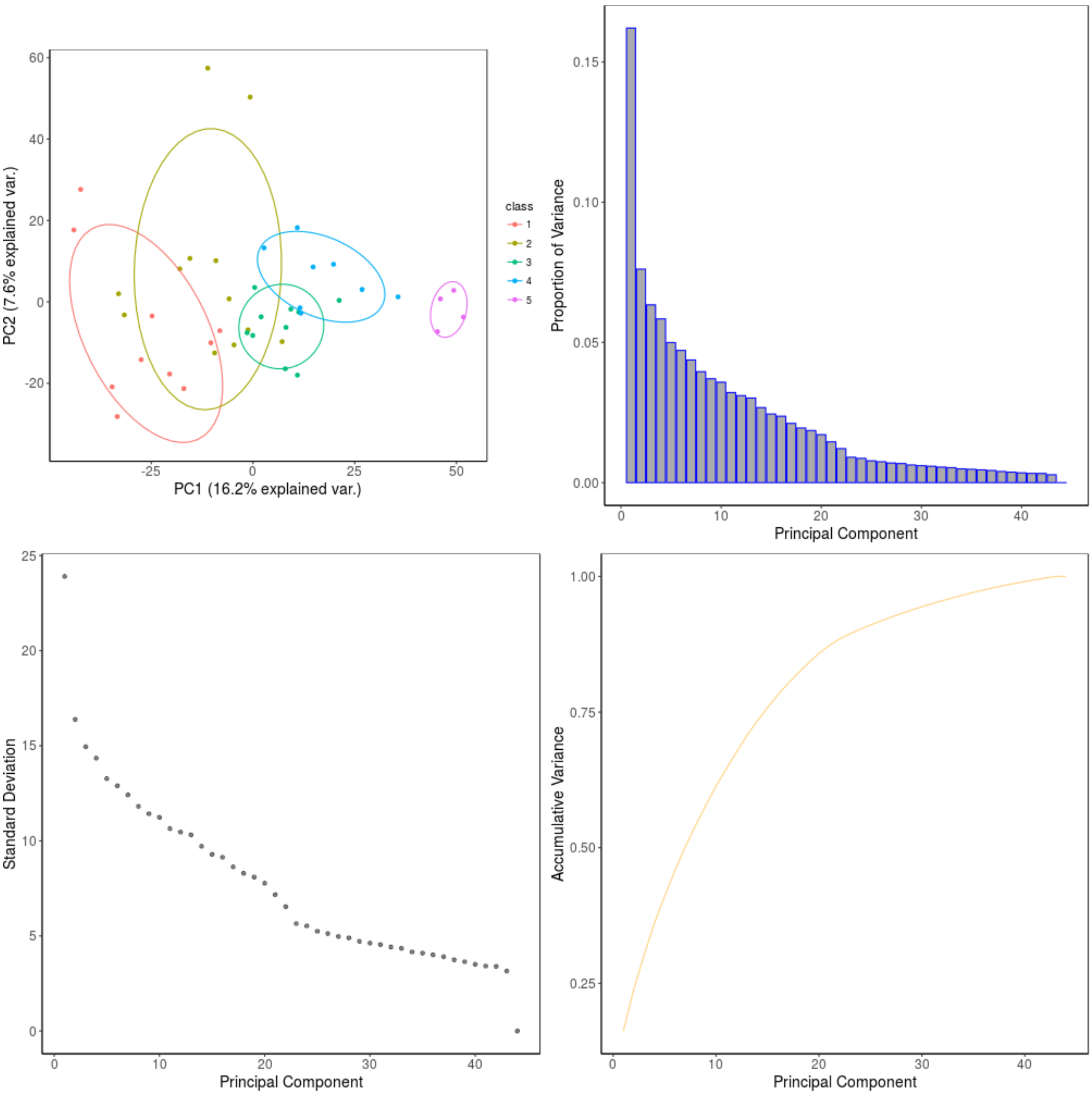

- Groups distribution on the first two Principal Components (PC1 and PC2) after to apply the FS workflow.

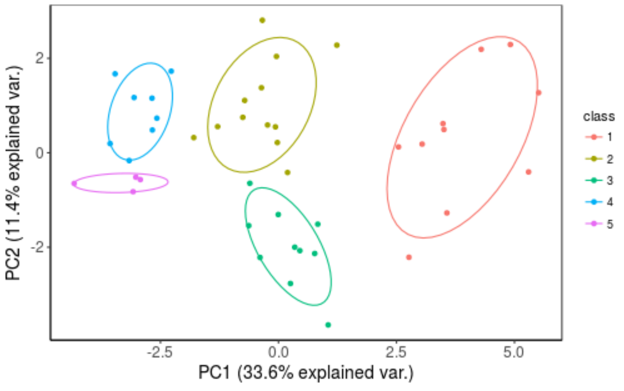

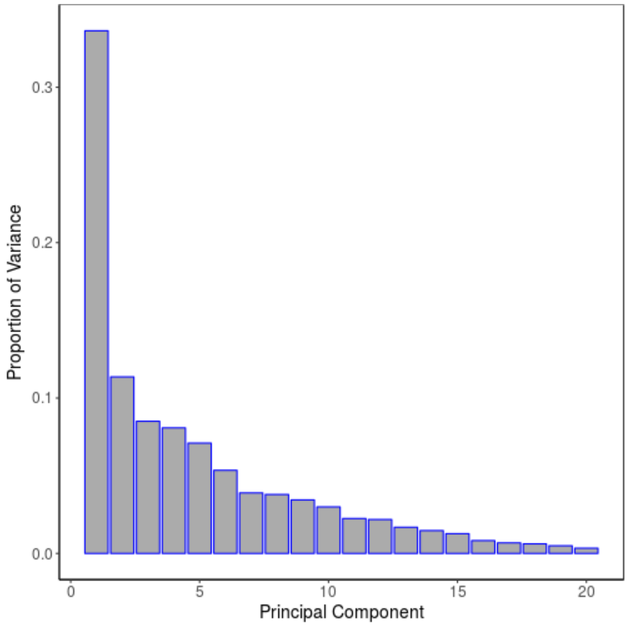

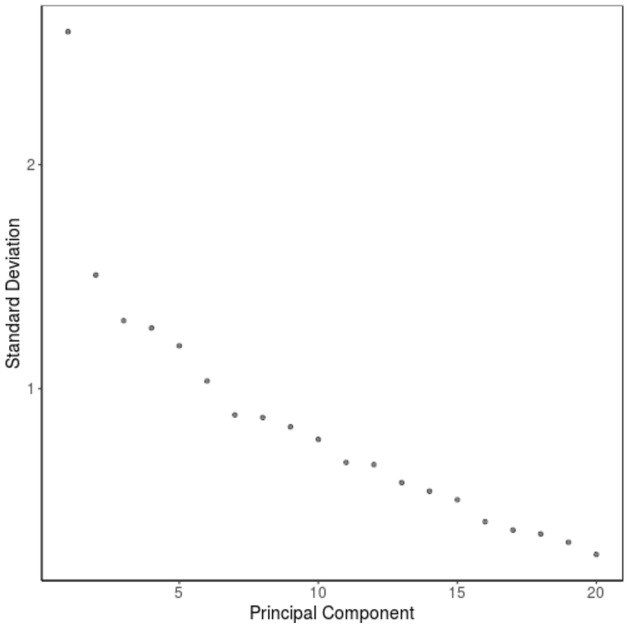

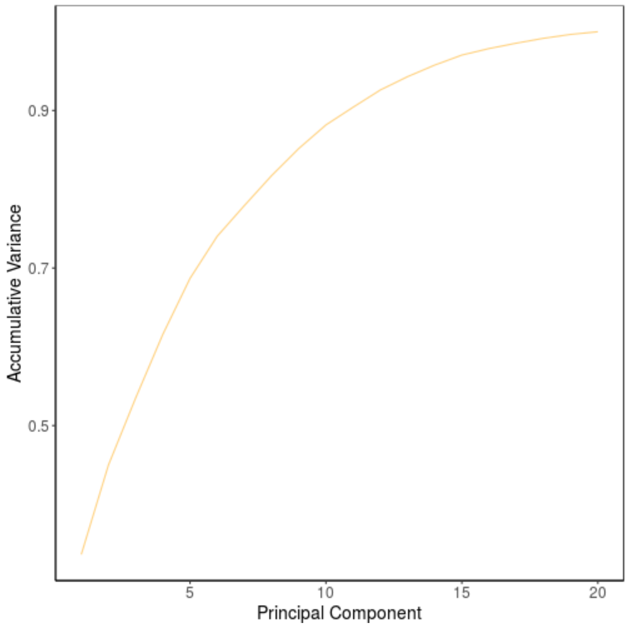

Feature Selection (FS) workflow report

May 18, 2017

Introduction

The report summarizing the Feature Selection pipeline results.

Feature Selection workflow

Univariate canonical correlation (X2) with Principal Component Analysis (PCA) follow by Recursive Feature Elimination (RFE) wrapped with Random Forest (RF).

Dataset

Triple-Negative Breast Cancer (TNBC) proteome. Label-free deep proteome analysis of 44 (samples and technical repliques) human breast specimens.

Summary stats from training phase

Table 1: Best model metrics from 10-folds cross-validation resampling.

Variables	Accuracy	Kappa	AccuracySD	KappaSD
1	0.68	0.5571	0.2227	0.3264
2	0.61	0.4708	0.2914	0.3758
3	0.7633	0.7	0.2471	0.3048
4	0.7917	0.7238	0.2013	0.2673
5	0.8467	0.7988	0.1798	0.2376
6	0.8667	0.8238	0.1851	0.2449
7	0.8467	0.7988	0.1798	0.2376
8	0.8667	0.8238	0.1851	0.2449
9	0.7967	0.7321	0.2007	0.2666
10	0.7967	0.7321	0.2007	0.2666
15	0.7967	0.7321	0.2007	0.2666
20	0.7967	0.7321	0.2007	0.2666
25	0.7467	0.6655	0.2066	0.2754
30	0.7717	0.6988	0.1877	0.2497
35	0.7717	0.6988	0.1877	0.2497
40	0.7717	0.6988	0.1877	0.2497
44	0.7967	0.7321	0.2007	0.2666

Summary stats from testing phase

Table 2: Classification metrics from twenty class-balanced and randomized runs.

run	Variables	Accuracy	Kappa	AccuracyPValue
1	15	1	1	2.216e-07
2	6	0.8462	0.803	9.42e-05
3	7	1	1	2.216e-07
4	15	0.7692	0.7068	0.000816
5	15	1	1	2.216e-07
6	35	1	1	2.216e-07
7	15	0.9231	0.8992	6.703e-06
8	9	0.9231	0.9015	6.703e-06
9	5	0.7692	0.6977	0.000816
10	4	0.8462	0.8	9.42e-05
11	20	0.9231	0.9008	6.703e-06
12	6	0.7692	0.7023	0.000816
13	8	0.9231	0.9008	6.703e-06
14	25	0.7692	0.7068	0.000816
15	8	0.8462	0.7969	9.42e-05
16	35	0.7692	0.7023	0.000816
17	8	0.8462	0.7969	9.42e-05
18	30	0.9231	0.8992	6.703e-06
**19**	**6**	**1**	**1**	**2.216e-07**
20	20	0.8462	0.803	9.42e-05

Accuracy__Mean	Accuracy__SD	Accuracy__Max
0.8846	0.08824	1

Workflow runtime

1.993 minutes

Plots

Visualization of the classification using PCA

- Groups distribution on the first two Principal Components (PC1 and PC2) from the original data (without apply any FS method).

```
## PCA plot not available for this FS workflow setting
```

- Groups distribution on the first two Principal Components (PC1 and PC2) after to apply the FS workflow.

```
## PCA plot not available for this FS workflow setting
```

Feature Selection (FS) workflow report

May 18, 2017

Introduction

The report summarizing the Feature Selection pipeline results.

Feature Selection workflow

Univariate canonical correlation (X2) with Multivariate Correlation filter (MC) follow by Recursive Feature Elimination (RFE) wrapped with Random Forest (RF).

Dataset

Transcriptomics analysis of left ventricles of mouse subjected to Isoproterenol challenge (GSE48760).

Summary stats from training phase

Table 1: Best model metrics from 10-folds cross-validation resampling.

Variables	Accuracy	Kappa	AccuracySD	KappaSD
1	0.7338	0.4673	0.1471	0.2946
2	0.8473	0.6953	0.1047	0.2092
3	0.8324	0.6664	0.1033	0.2056
4	0.8396	0.6807	0.1076	0.2141
5	0.833	0.6671	0.0964	0.1918
6	0.8401	0.6814	0.1213	0.2418
7	0.8692	0.7388	0.1003	0.2006
8	0.8621	0.7245	0.1142	0.2283
9	0.8401	0.6814	0.1346	0.2685
10	0.8544	0.7099	0.1179	0.2348
15	0.8473	0.6956	0.11	0.219
20	0.8473	0.6956	0.1333	0.2658
25	0.8544	0.7099	0.1179	0.2348
30	0.8544	0.7099	0.1179	0.2348
35	0.8544	0.7099	0.1179	0.2348
40	0.8544	0.7099	0.1179	0.2348
45	0.8544	0.7099	0.1179	0.2348
50	0.8544	0.7099	0.1179	0.2348
60	0.8615	0.7242	0.1051	0.2091
70	0.8615	0.7242	0.1051	0.2091
80	0.8687	0.7385	0.1124	0.2235
90	0.8687	0.7385	0.1124	0.2235
100	0.8615	0.7242	0.1249	0.2487
482	0.8687	0.7385	0.1124	0.2235

Summary stats from testing phase

Table 2: Classification metrics from twenty class-balanced and randomized runs.

run	Variables	Accuracy	Kappa	AccuracyPValue
**1**	**7**	**0.9275**	**0.8551**	**4.82e-14**
2	40	0.8986	0.7968	4.536e-12
3	6	0.8696	0.739	2.345e-10
4	482	0.8986	0.7971	4.536e-12
5	7	0.8841	0.7685	3.485e-11
6	20	0.8841	0.7683	3.485e-11
7	25	0.8406	0.682	7.46e-09
8	6	0.913	0.8259	5.088e-13
9	80	0.8116	0.6239	1.572e-07
10	60	0.9275	0.8549	4.82e-14
11	9	0.8261	0.6524	3.592e-08
12	80	0.8551	0.7103	1.398e-09
13	15	0.8986	0.7973	4.536e-12
14	40	0.8696	0.7396	2.345e-10
15	9	0.8551	0.7101	1.398e-09
16	40	0.8406	0.6815	7.46e-09
17	80	0.913	0.8261	5.088e-13
18	482	0.913	0.8262	5.088e-13
19	50	0.8696	0.7398	2.345e-10
20	482	0.913	0.8259	5.088e-13

Accuracy_Mean	Accuracy_SD	Accuracy_Max
0.8804	0.03419	0.9275

Workflow runtime

8.885 minutes

Plots

Visualization of the classification using PCA

- Groups distribution on the first two Principal Components (PC1 and PC2) from the original data (without apply any FS method).

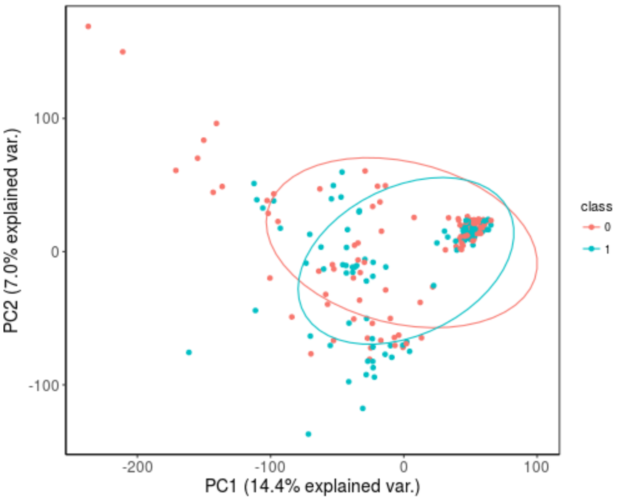

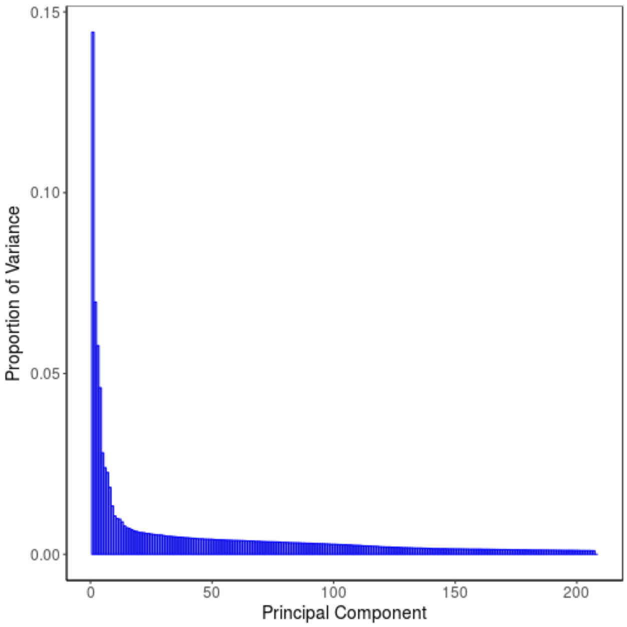

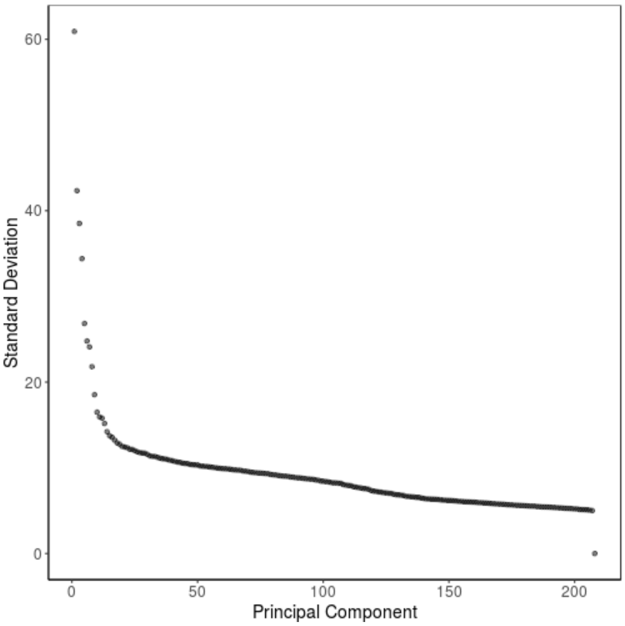

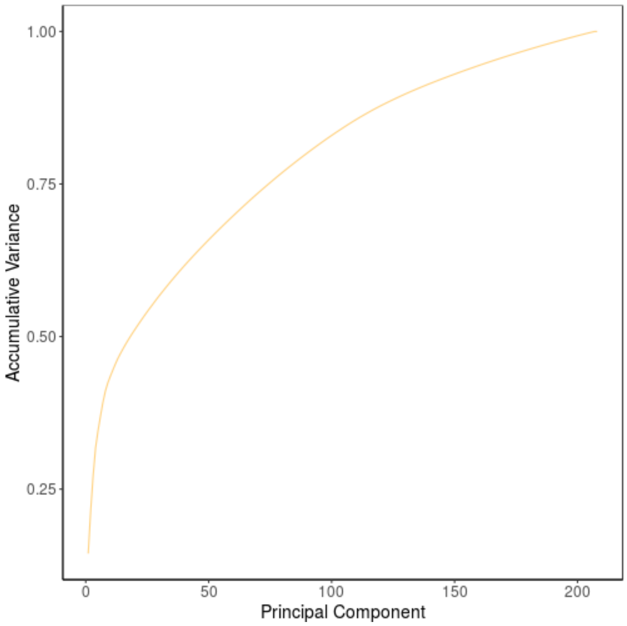

- Groups distribution on the first two Principal Components (PC1 and PC2) after to apply the FS workflow.

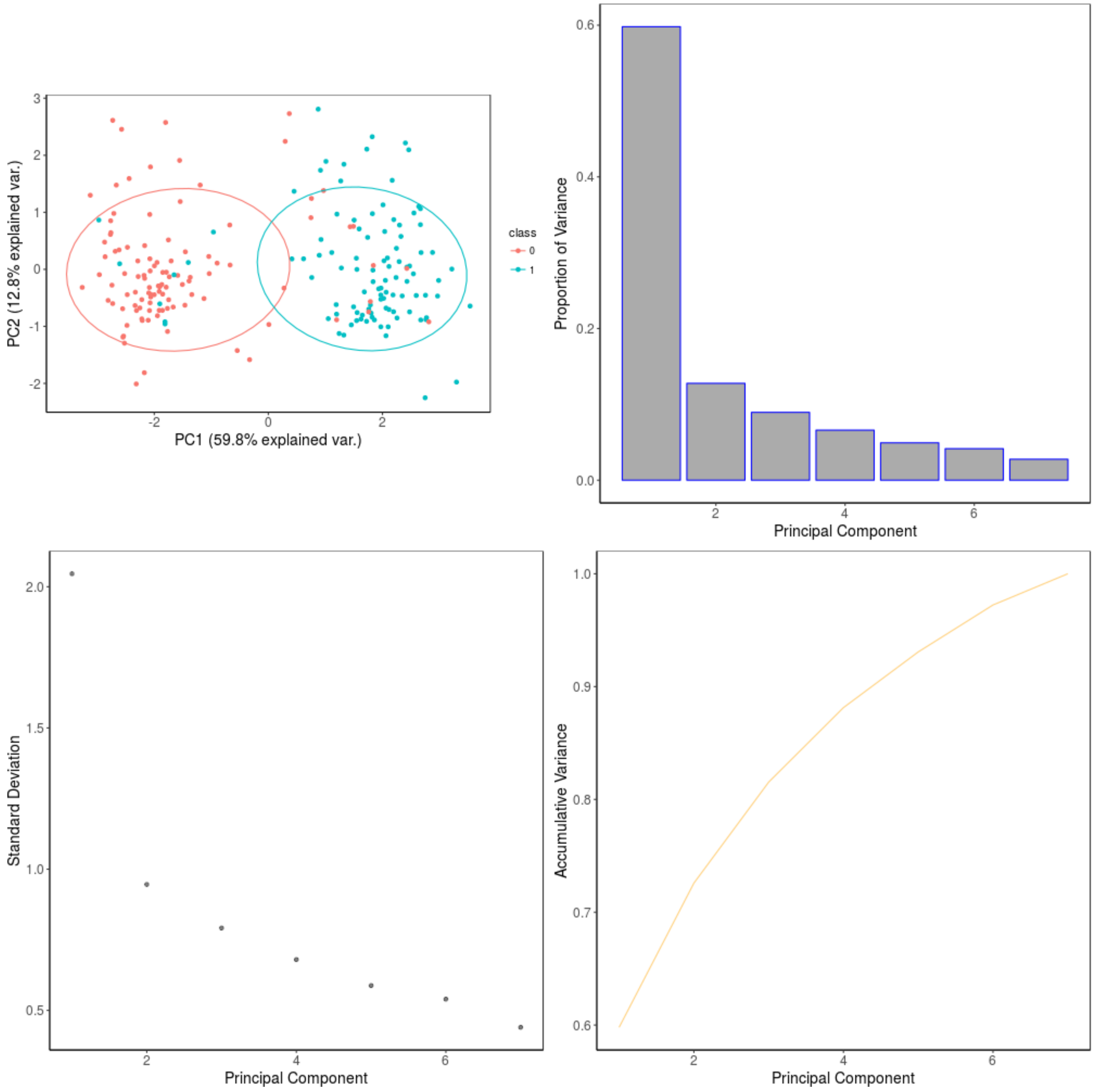

Feature Selection (FS) workflow report

May 18, 2017

Introduction

The report summarizing the Feature Selection pipeline results.

Feature Selection workflow

Univariate canonical correlation (X2) with Principal Component Analysis (PCA) follow by Recursive Feature Elimination (RFE) wrapped with Random Forest (RF).

Dataset

Transcriptomics analysis of left ventricles of mouse subjected to Isoproterenol challenge (GSE48760).

Summary stats from training phase

Table 1: Best model metrics from 10-folds cross-validation resampling.

Variables	Accuracy	Kappa	AccuracySD	KappaSD
1	0.7835	0.5655	0.07023	0.1423
2	0.8775	0.7548	0.03542	0.07103
3	0.8478	0.6949	0.06799	0.1353
4	0.8918	0.7827	0.03894	0.07873
5	0.906	0.8119	0.0364	0.07313
6	0.8846	0.769	0.0696	0.1393
7	0.8846	0.769	0.05076	0.1017
8	0.8918	0.7833	0.05148	0.1031
9	0.8918	0.7833	0.05148	0.1031
10	0.8989	0.7976	0.03844	0.07715
15	0.8923	0.7847	0.0501	0.1003
20	0.8846	0.7698	0.03798	0.07537
25	0.8846	0.769	0.03798	0.07617
30	0.8769	0.7541	0.06185	0.1232
35	0.8775	0.7548	0.05935	0.1188
40	0.8626	0.7255	0.06501	0.1296
45	0.8918	0.7833	0.05148	0.1031
50	0.8775	0.7548	0.03542	0.07103
60	0.8846	0.769	0.03798	0.07617
70	0.8775	0.7548	0.03542	0.07103
80	0.8846	0.769	0.03798	0.07617
90	0.8846	0.7698	0.03798	0.07537
100	0.8775	0.7548	0.03542	0.07103
208	0.8555	0.7113	0.06945	0.1385

Summary stats from testing phase

Table 2: Classification metrics from twenty class-balanced and randomized runs.

run	Variables	Accuracy	Kappa	AccuracyPValue
1	35	0.8696	0.7394	2.345e-10
2	25	0.8841	0.7679	3.485e-11
3	30	0.8696	0.7396	2.345e-10
4	50	0.8551	0.7106	1.398e-09
5	25	0.8986	0.7973	4.536e-12
6	5	0.8986	0.7973	4.536e-12
7	8	0.8406	0.6812	7.46e-09
8	30	0.8696	0.7394	2.345e-10
9	9	0.8551	0.7098	1.398e-09
10	9	0.8261	0.6533	3.592e-08
**11**	**5**	**0.9275**	**0.8551**	**4.82e-14**
12	6	0.8551	0.7103	1.398e-09
13	100	0.913	0.8261	5.088e-13
14	5	0.8551	0.7098	1.398e-09
15	80	0.8406	0.682	7.46e-09
16	9	0.8841	0.7681	3.485e-11
17	50	0.8841	0.7681	3.485e-11
18	7	0.8696	0.7392	2.345e-10
19	3	0.8986	0.7973	4.536e-12
20	2	0.8261	0.653	3.592e-08

Accuracy_Mean	Accuracy_SD	Accuracy_Max
0.871	0.02778	0.9275

Workflow runtime

8.492 minutes

Plots

Visualization of the classification using PCA

- Groups distribution on the first two Principal Components (PC1 and PC2) from the original data (without apply any FS method).

```
## PCA plot not available for this FS workflow setting
```

- Groups distribution on the first two Principal Components (PC1 and PC2) after to apply the FS workflow.

```
## PCA plot not available for this FS workflow setting
```

Feature Selection (FS) workflow report

May 18, 2017

Introduction

The report summarizing the Feature Selection pipeline results.

Feature Selection workflow

Univariate canonical correlation (X2) with Multivariate Correlation filter (MC) follow by Recursive Feature Elimination (RFE) wrapped with Random Forest (RF).

Dataset

Expression data from normal and prostate tumor tissues (GSE6919_GPL8300).

Summary stats from training phase

Table 1: Best model metrics from 10-folds cross-validation resampling.

Variables	Accuracy	Kappa	AccuracySD	KappaSD
1	0.5584	0.3525	0.1223	0.165
2	0.4863	0.2349	0.1062	0.17
3	0.5305	0.3069	0.1674	0.2532
4	0.5235	0.2879	0.1565	0.2329
5	0.5591	0.3379	0.1273	0.1922
6	0.5703	0.3482	0.1361	0.2158
7	0.5863	0.3779	0.1014	0.1541
8	0.5876	0.3824	0.1182	0.1671
9	0.5862	0.3831	0.1064	0.1513
10	0.5612	0.341	0.1144	0.1709
15	0.6015	0.3971	0.136	0.2083
20	0.6568	0.4783	0.1345	0.2087
25	0.6363	0.4502	0.1168	0.1775
30	0.6545	0.4817	0.1096	0.1685
35	0.6445	0.4657	0.1653	0.2565
40	0.653	0.4783	0.1276	0.198
45	0.669	0.502	0.13	0.201
50	0.6886	0.5314	0.1306	0.1977
60	0.6257	0.442	0.1247	0.1795
70	0.6691	0.5065	0.08924	0.1301
80	0.6608	0.492	0.06148	0.09258
90	0.6448	0.4676	0.07477	0.1111
100	0.6706	0.5072	0.08379	0.1266
349	0.6796	0.5201	0.09817	0.1474

Summary stats from testing phase

Table 2: Classification metrics from twenty class-balanced and randomized runs.

run	Variables	Accuracy	Kappa	AccuracyPValue
1	45	0.6786	0.5186	4.002e-06
2	50	0.7143	0.5858	2.677e-07
3	90	0.7143	0.5748	2.677e-07
**4**	**50**	**0.8393**	**0.7658**	**1.182e-12**
5	40	0.7679	0.652	2.45e-09
6	50	0.6964	0.5545	1.078e-06
7	90	0.7321	0.6093	6.116e-08
8	70	0.6786	0.5156	4.002e-06
9	60	0.6964	0.5572	1.078e-06
10	60	0.625	0.4308	0.0001297
11	80	0.7321	0.6069	6.116e-08
12	35	0.6429	0.4786	4.381e-05
13	40	0.7143	0.5833	2.677e-07
14	20	0.7143	0.5833	2.677e-07
15	20	0.7143	0.5782	2.677e-07
16	100	0.6429	0.4791	4.381e-05
17	90	0.6964	0.558	1.078e-06
18	80	0.7857	0.6791	4.256e-10
19	70	0.6607	0.509	1.375e-05
20	100	0.7143	0.5807	2.677e-07

Accuracy_Mean	Accuracy_SD	Accuracy_Max
0.708	0.05027	0.8393

Workflow runtime

8.269 minutes

Plots

Visualization of the classification using PCA

- Groups distribution on the first two Principal Components (PC1 and PC2) from the original data (without apply any FS method).

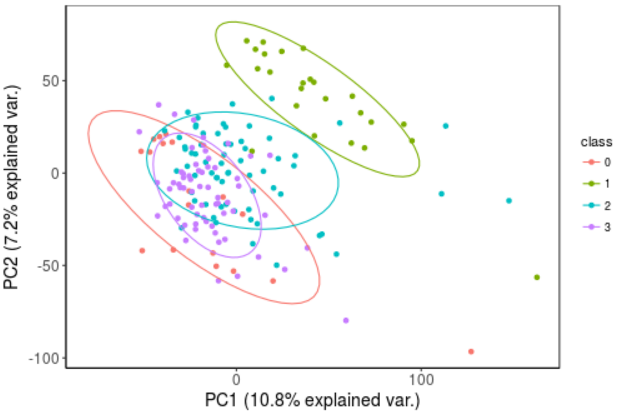

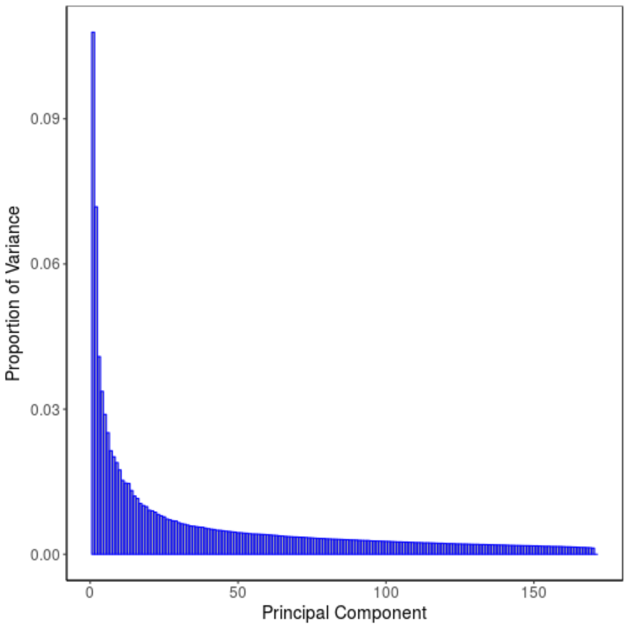

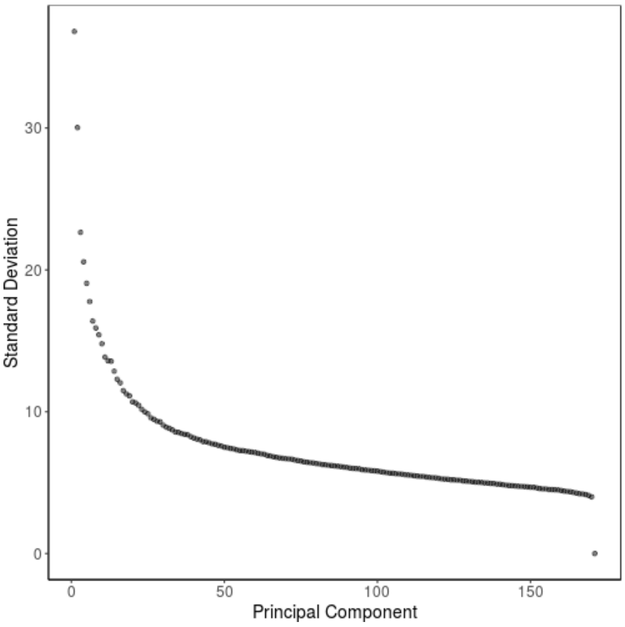

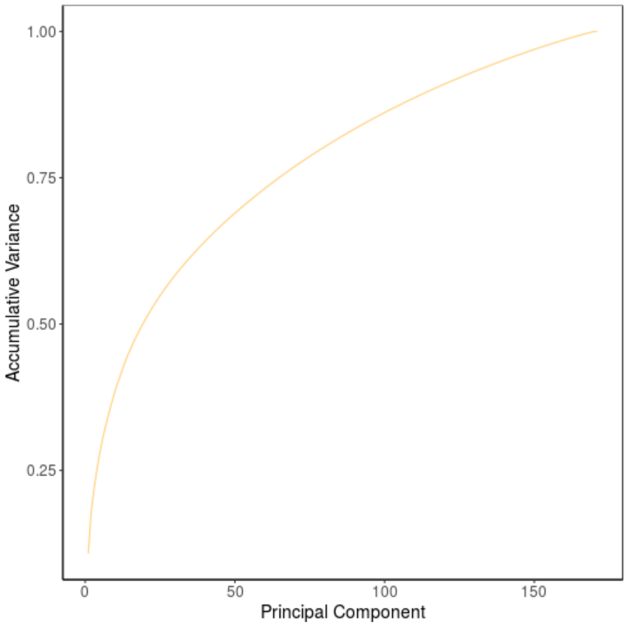

- Groups distribution on the first two Principal Components (PC1 and PC2) after to apply the FS workflow.

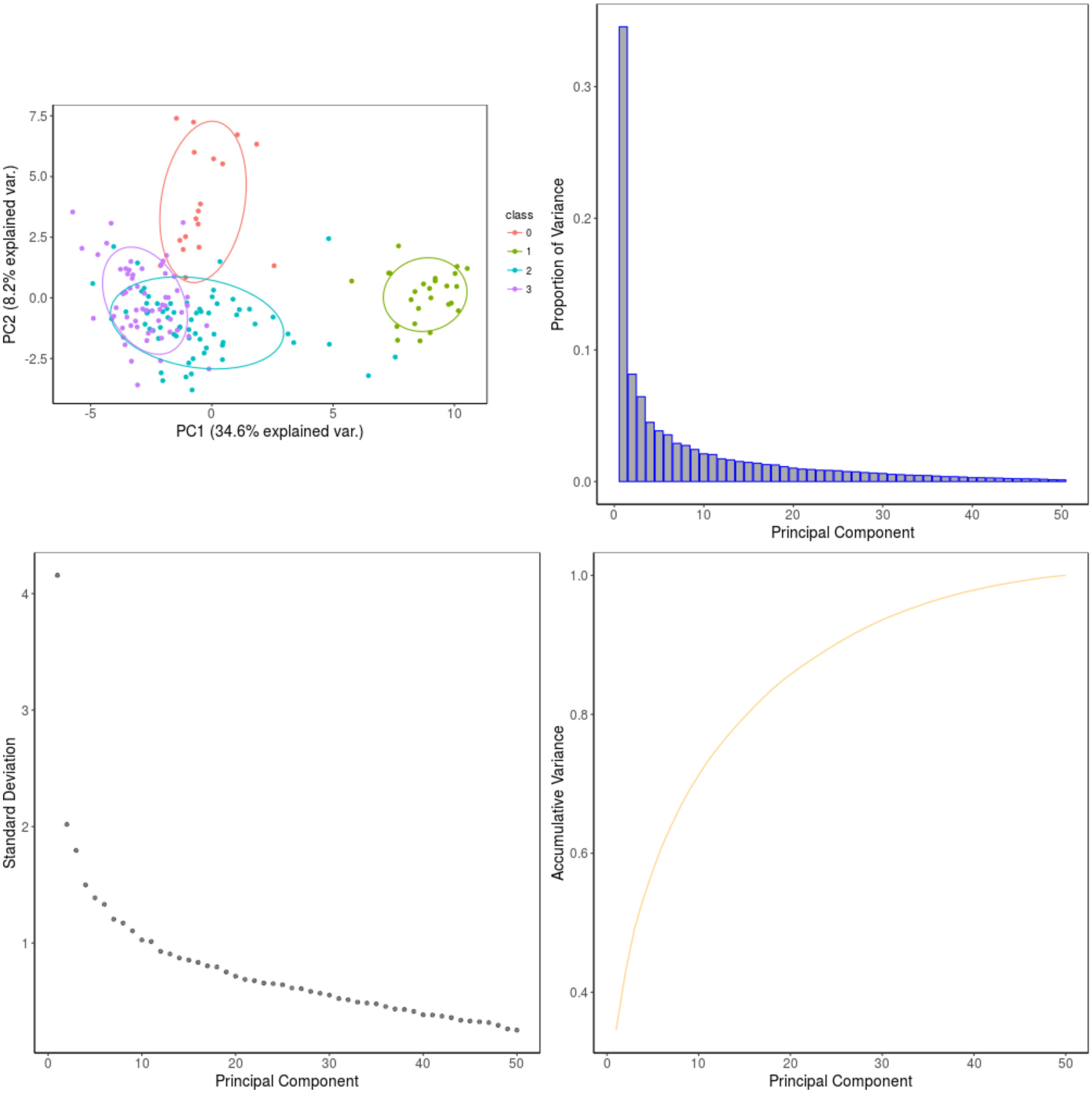

Feature Selection (FS) workflow report

May 18, 2017

Introduction

The report summarizing the Feature Selection pipeline results.

Feature Selection workflow

Univariate canonical correlation (X2) with Principal Component Analysis (PCA) follow by Recursive Feature Elimination (RFE) wrapped with Random Forest (RF).

Dataset

Expression data from normal and prostate tumor tissues (GSE6919_GPL8300).

Summary stats from training phase

Table 1: Best model metrics from 10-folds cross-validation resampling.

Variables	Accuracy	Kappa	AccuracySD	KappaSD
1	0.6135	0.4221	0.09185	0.1321
2	0.6161	0.4294	0.1087	0.1592
3	0.6032	0.4093	0.1475	0.2238
4	0.6329	0.4596	0.1332	0.1955
5	0.6243	0.4408	0.1126	0.1689
6	0.7121	0.573	0.1087	0.1611
7	0.6925	0.5449	0.1342	0.199
8	0.6908	0.5409	0.1648	0.2459
9	0.7173	0.5785	0.1378	0.2052
10	0.6908	0.5414	0.1807	0.2688
15	0.7014	0.556	0.1521	0.2242
20	0.6937	0.5423	0.1574	0.2326
25	0.6869	0.5328	0.1444	0.2104
30	0.7114	0.5677	0.1375	0.2036
35	0.7212	0.5838	0.153	0.2243
40	0.6912	0.5372	0.1639	0.2422
45	0.6662	0.4994	0.1919	0.2852
50	0.6659	0.4925	0.1849	0.2777
60	0.6572	0.4849	0.1755	0.2624
70	0.639	0.4552	0.153	0.2278
80	0.6238	0.428	0.1674	0.2516
90	0.5993	0.389	0.1468	0.2193
100	0.6193	0.4229	0.1939	0.285
171	0.5192	0.2528	0.1697	0.2588

Summary stats from testing phase

Table 2: Classification metrics from twenty class-balanced and randomized runs.

run	Variables	Accuracy	Kappa	AccuracyPValue
1	4	0.6429	0.4832	4.381e-05
2	10	0.7321	0.61	6.116e-08
3	70	0.6964	0.5454	1.078e-06
**4**	**35**	**0.7679**	**0.6576**	**2.45e-09**
5	5	0.6964	0.549	1.078e-06
6	4	0.75	0.6286	1.281e-08
7	8	0.7143	0.5861	2.677e-07
8	20	0.6607	0.5	1.375e-05
9	3	0.5714	0.3705	0.002204
10	6	0.6607	0.5035	1.375e-05
11	30	0.6964	0.5572	1.078e-06
12	8	0.6964	0.5549	1.078e-06
13	9	0.6964	0.5611	1.078e-06
14	15	0.6786	0.5191	4.002e-06
15	5	0.6964	0.5584	1.078e-06
16	6	0.75	0.6338	1.281e-08
17	10	0.6607	0.5051	1.375e-05
18	2	0.6964	0.5611	1.078e-06
19	10	0.7143	0.5815	2.677e-07
20	20	0.7143	0.5811	2.677e-07

Accuracy_Mean	Accuracy_SD	Accuracy_Max
0.6946	0.04332	0.7679

Workflow runtime

8.498 minutes

Plots

Visualization of the classification using PCA

- Groups distribution on the first two Principal Components (PC1 and PC2) from the original data (without apply any FS method).

```
## PCA plot not available for this FS workflow setting
```

- Groups distribution on the first two Principal Components (PC1 and PC2) after to apply the FS workflow.

```
## PCA plot not available for this FS workflow setting
```

Feature Selection (FS) workflow report

May 18, 2017

Introduction

The report summarizing the Feature Selection pipeline results.

Feature Selection workflow

Univariate canonical correlation (X2) with Multivariate Correlation filter (MC) follow by Recursive Feature Elimination (RFE) wrapped with Random Forest (RF).

Dataset

Expression data from normal and prostate tumor tissues (GSE6919_GPL92).

Summary stats from training phase

Table 1: Best model metrics from 10-folds cross-validation resampling.

Variables	Accuracy	Kappa	AccuracySD	KappaSD
1	0.5055	0.3003	0.1167	0.1974
2	0.5327	0.3086	0.1059	0.1543
3	0.6298	0.4504	0.109	0.1578
4	0.6282	0.45	0.07632	0.1016
5	0.683	0.5307	0.1166	0.1582
6	0.648	0.4827	0.1269	0.1706
7	0.6779	0.527	0.1806	0.2479
8	0.6764	0.5214	0.1609	0.2228
9	0.6574	0.4951	0.1641	0.2288
10	0.6748	0.5249	0.1665	0.2278
15	0.6738	0.5175	0.1492	0.2082
20	0.692	0.5366	0.09322	0.1352
25	0.6994	0.5518	0.09171	0.1255
30	0.6629	0.4957	0.1039	0.1496
35	0.6636	0.4962	0.1235	0.1783
40	0.6738	0.5119	0.08651	0.1169
45	0.7027	0.5571	0.1182	0.1566
50	0.7027	0.5599	0.1115	0.14
60	0.6936	0.5428	0.113	0.1527
70	0.6853	0.5307	0.1543	0.2219
80	0.702	0.5524	0.09934	0.1365
90	0.6838	0.5261	0.1102	0.1486
100	0.6573	0.4886	0.1423	0.1921
327	0.6952	0.5449	0.1619	0.2312

Summary stats from testing phase

Table 2: Classification metrics from twenty class-balanced and randomized runs.

run	Variables	Accuracy	Kappa	AccuracyPValue
1	100	0.7273	0.5846	8.87e-07
2	40	0.6182	0.4231	0.0008835
3	100	0.7455	0.6119	2.106e-07
4	60	0.6727	0.5015	3.979e-05
5	80	0.6182	0.4116	0.0008835
6	50	0.7273	0.5881	8.87e-07
7	60	0.7273	0.5821	8.87e-07
8	100	0.6545	0.4746	0.0001205
9	327	0.7091	0.5535	3.424e-06
10	327	0.7455	0.6158	2.106e-07
11	30	0.6727	0.503	3.979e-05
12	327	0.7455	0.6146	2.106e-07
**13**	**45**	**0.7818**	**0.6697**	**8.988e-09**
14	25	0.6364	0.4514	0.0003385
15	327	0.6182	0.419	0.0008835
16	100	0.6	0.3913	0.002147
17	50	0.7818	0.6713	8.988e-09
18	327	0.7455	0.6101	2.106e-07
19	45	0.6909	0.536	1.215e-05
20	327	0.6545	0.4703	0.0001205

Accuracy__Mean	Accuracy__SD	Accuracy__Max
0.6936	0.05758	0.7818

Workflow runtime

8.189 minutes

Plots

Visualization of the classification using PCA

- Groups distribution on the first two Principal Components (PC1 and PC2) from the original data (without apply any FS method).

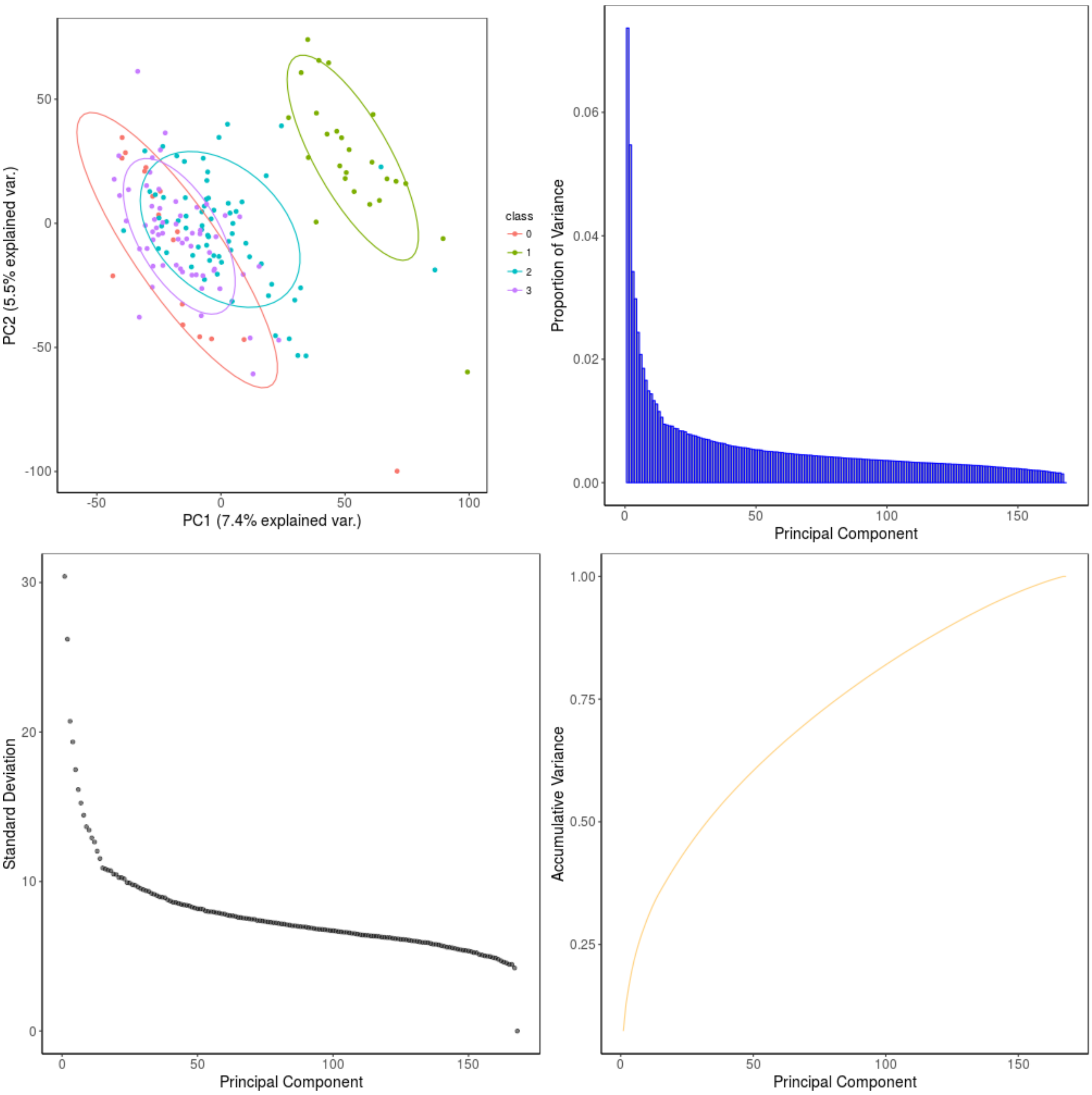

- Groups distribution on the first two Principal Components (PC1 and PC2) after to apply the FS workflow.

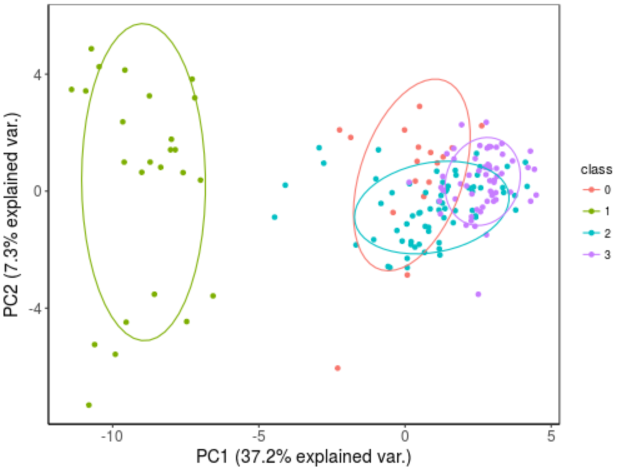

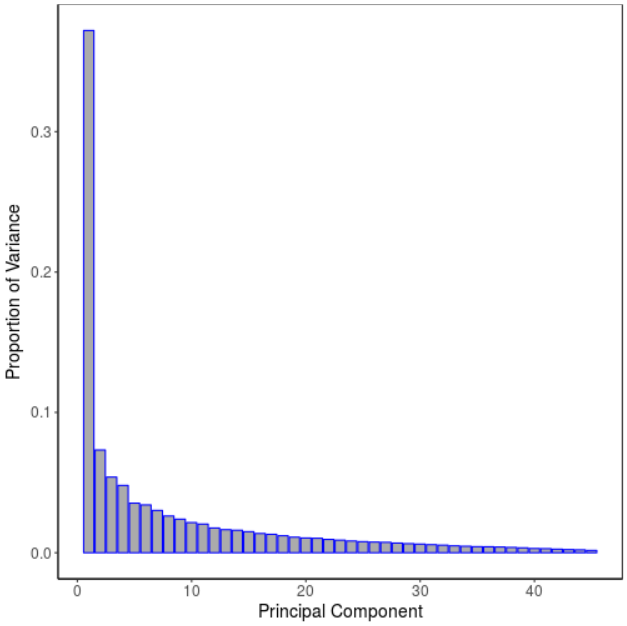

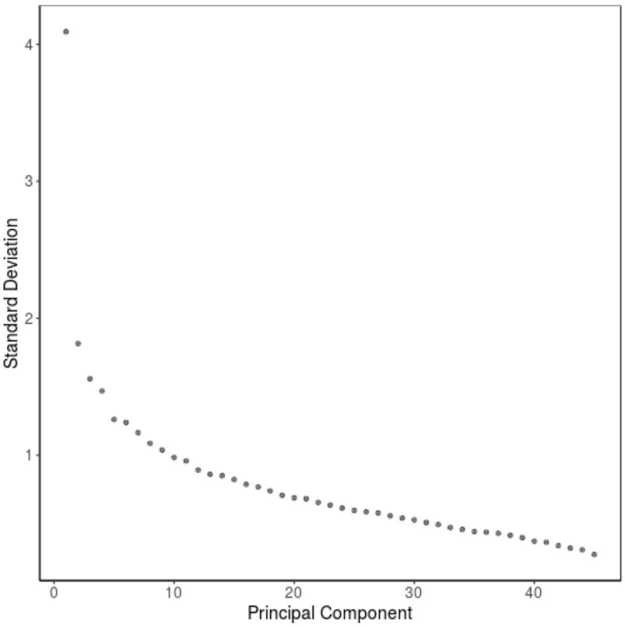

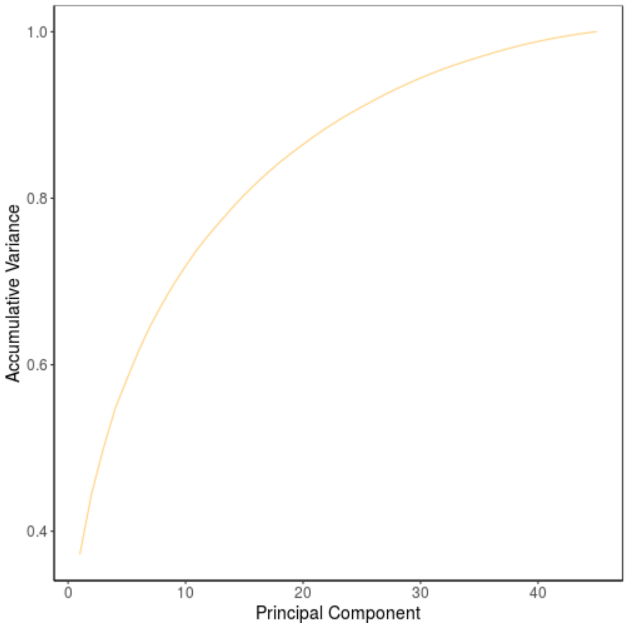

Feature Selection (FS) workflow report

May 18, 2017

Introduction

The report summarizing the Feature Selection pipeline results.

Feature Selection workflow

Univariate canonical correlation (X2) with Principal Component Analysis (PCA) follow by Recursive Feature Elimination (RFE) wrapped with Random Forest (RF).

Dataset

Expression data from normal and prostate tumor tissues (GSE6919_GPL92).

Summary stats from training phase

Table 1: Best model metrics from 10-folds cross-validation resampling.

Variables	Accuracy	Kappa	AccuracySD	KappaSD
1	0.4917	0.2599	0.1455	0.2233
2	0.6152	0.4309	0.1671	0.2364
3	0.6597	0.4956	0.1872	0.2755
4	0.6894	0.5492	0.1776	0.2536
5	0.7045	0.5686	0.1834	0.2653
6	0.6515	0.4875	0.1811	0.2627
7	0.6083	0.4168	0.1626	0.2375
8	0.6788	0.5208	0.1383	0.2053
9	0.6788	0.5232	0.1766	0.2563
10	0.7038	0.5586	0.1512	0.2272
15	0.6773	0.5155	0.1155	0.1823
20	0.6508	0.4722	0.1192	0.1806
25	0.6797	0.521	0.1048	0.1512
30	0.6956	0.5411	0.1262	0.1856
35	0.6789	0.513	0.09155	0.1344
40	0.6698	0.4952	0.1377	0.2054
45	0.6515	0.47	0.1161	0.1715
50	0.6076	0.4035	0.1253	0.1823
60	0.6523	0.4719	0.1208	0.1752
70	0.6674	0.4929	0.151	0.2286
80	0.615	0.4135	0.1665	0.2504
90	0.5983	0.3853	0.1434	0.2186
100	0.6432	0.4568	0.1361	0.204
168	0.6165	0.4093	0.1455	0.2202

Summary stats from testing phase

Table 2: Classification metrics from twenty class-balanced and randomized runs.

run	Variables	Accuracy	Kappa	AccuracyPValue
**1**	**5**	**0.8**	**0.7027**	**1.599e-09**
2	9	0.7091	0.5578	3.424e-06
3	5	0.7091	0.5578	3.424e-06
4	6	0.6364	0.4481	0.0003385
5	4	0.6727	0.513	3.979e-05
6	3	0.6909	0.5394	1.215e-05
7	8	0.6909	0.5457	1.215e-05
8	25	0.7091	0.565	3.424e-06
9	10	0.7273	0.5966	8.87e-07
10	60	0.6909	0.5227	1.215e-05
11	15	0.8	0.7052	1.599e-09
12	7	0.7455	0.6163	2.106e-07
13	30	0.6909	0.5273	1.215e-05
14	8	0.7455	0.6131	2.106e-07
15	5	0.7818	0.6749	8.988e-09
16	10	0.6727	0.5254	3.979e-05
17	45	0.7636	0.6407	4.565e-08
18	9	0.7273	0.5869	8.87e-07
19	25	0.6909	0.5396	1.215e-05
20	10	0.7455	0.6282	2.106e-07

Accuracy__Mean	Accuracy__SD	Accuracy__Max
0.72	0.04391	0.8

Workflow runtime

9.108 minutes

Plots

Visualization of the classification using PCA

- Groups distribution on the first two Principal Components (PC1 and PC2) from the original data (without apply any FS method).

```
## PCA plot not available for this FS workflow setting
```

- Groups distribution on the first two Principal Components (PC1 and PC2) after to apply the FS workflow.

```
## PCA plot not available for this FS workflow setting
```

Feature Selection (FS) workflow report

May 18, 2017

Introduction

The report summarizing the Feature Selection pipeline results.

Feature Selection workflow

Univariate canonical correlation (X2) with Multivariate Correlation filter (MC) follow by Recursive Feature Elimination (RFE) wrapped with Random Forest (RF).

Dataset

Expression data from normal and prostate tumor tissues (GSE6919_GPL93).

Summary stats from training phase

Table 1: Best model metrics from 10-folds cross-validation resampling.

Variables	Accuracy	Kappa	AccuracySD	KappaSD
1	0.4553	0.208	0.1297	0.1822
2	0.5061	0.2678	0.09259	0.1535
3	0.5654	0.3547	0.1292	0.2117
4	0.5576	0.3413	0.1028	0.1608
5	0.6018	0.4033	0.1006	0.1717
6	0.5873	0.3829	0.09309	0.1501
7	0.5762	0.3653	0.1034	0.1724
8	0.5684	0.3577	0.09698	0.1601
9	0.5948	0.3999	0.09452	0.158
10	0.5669	0.3597	0.08491	0.1386
15	0.5396	0.3116	0.1263	0.1936
20	0.5623	0.3446	0.1269	0.2078
25	0.638	0.4602	0.1189	0.1801
30	0.6052	0.415	0.11	0.1648
35	0.6237	0.4477	0.1118	0.1584
40	0.6502	0.4791	0.1162	0.1706
45	0.6616	0.503	0.1195	0.1739
50	0.6954	0.5471	0.105	0.1645
60	0.6841	0.5314	0.09131	0.1379
70	0.6904	0.5355	0.1151	0.1932
80	0.6841	0.5281	0.08243	0.128
90	0.6778	0.5212	0.06956	0.09924
100	0.6674	0.5002	0.06359	0.1111
459	0.6788	0.5205	0.08209	0.1198

Summary stats from testing phase

Table 2: Classification metrics from twenty class-balanced and randomized runs.

run	Variables	Accuracy	Kappa	AccuracyPValue
1	35	0.7358	0.6099	5.533e-07
2	459	0.6792	0.5148	2.884e-05
3	45	0.6226	0.4329	0.0007184
4	80	0.6981	0.5504	8.413e-06
5	70	0.7358	0.6066	5.533e-07
6	40	0.6604	0.4871	9.108e-05
7	459	0.6604	0.486	9.108e-05
8	70	0.6981	0.5361	8.413e-06
9	459	0.6604	0.4826	9.108e-05
10	60	0.717	0.5789	2.255e-06
11	100	0.6792	0.5225	2.884e-05
12	90	0.717	0.5771	2.255e-06
13	459	0.7358	0.6024	5.533e-07
14	459	0.7358	0.5959	5.533e-07
15	90	0.717	0.5803	2.255e-06
16	10	0.717	0.578	2.255e-06
17	70	0.6604	0.4832	9.108e-05
18	90	0.6415	0.4606	0.0002658
**19**	**50**	**0.7547**	**0.6284**	**1.238e-07**
20	100	0.6226	0.4307	0.0007184

Accuracy_Mean	Accuracy_SD	Accuracy_Max
0.6925	0.04019	0.7547

Workflow runtime

8.664 minutes

Plots

Visualization of the classification using PCA

- Groups distribution on the first two Principal Components (PC1 and PC2) from the original data (without apply any FS method).

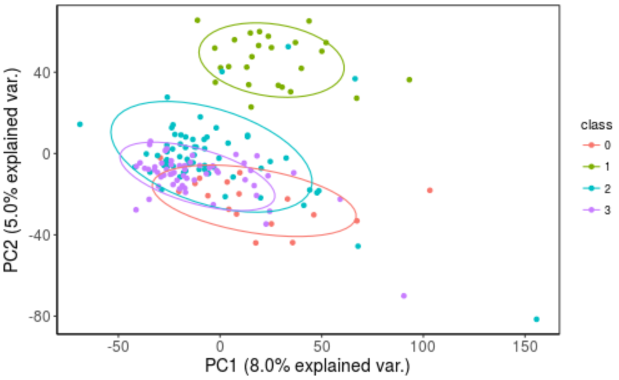

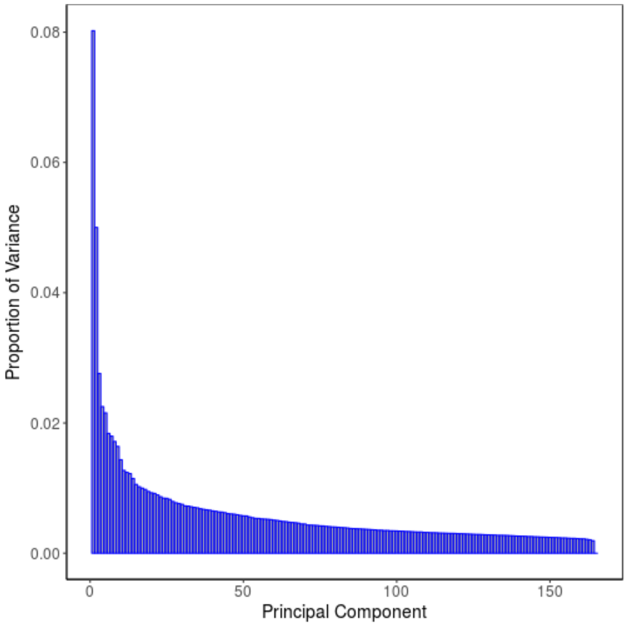

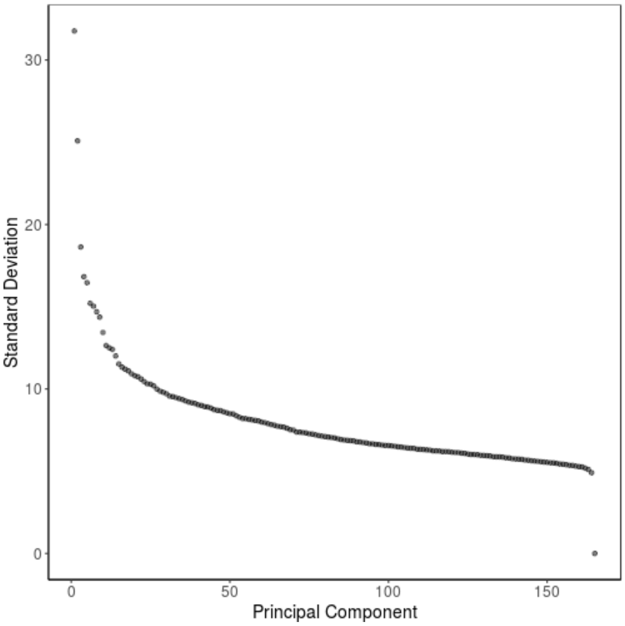

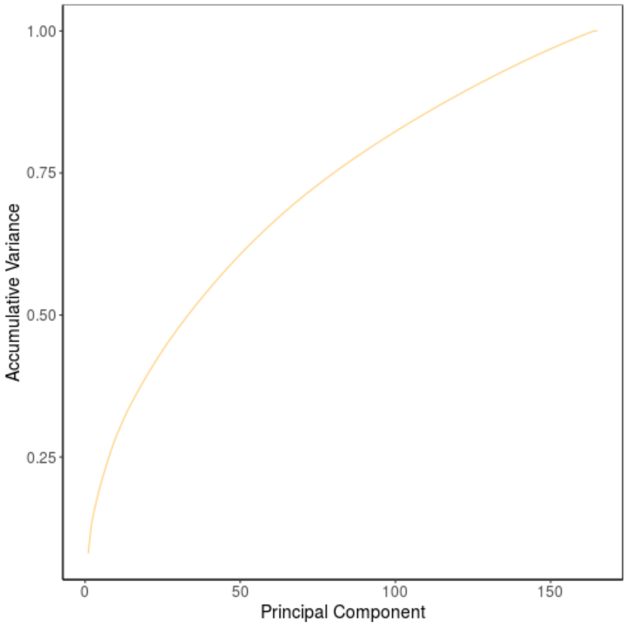

- Groups distribution on the first two Principal Components (PC1 and PC2) after to apply the FS workflow.

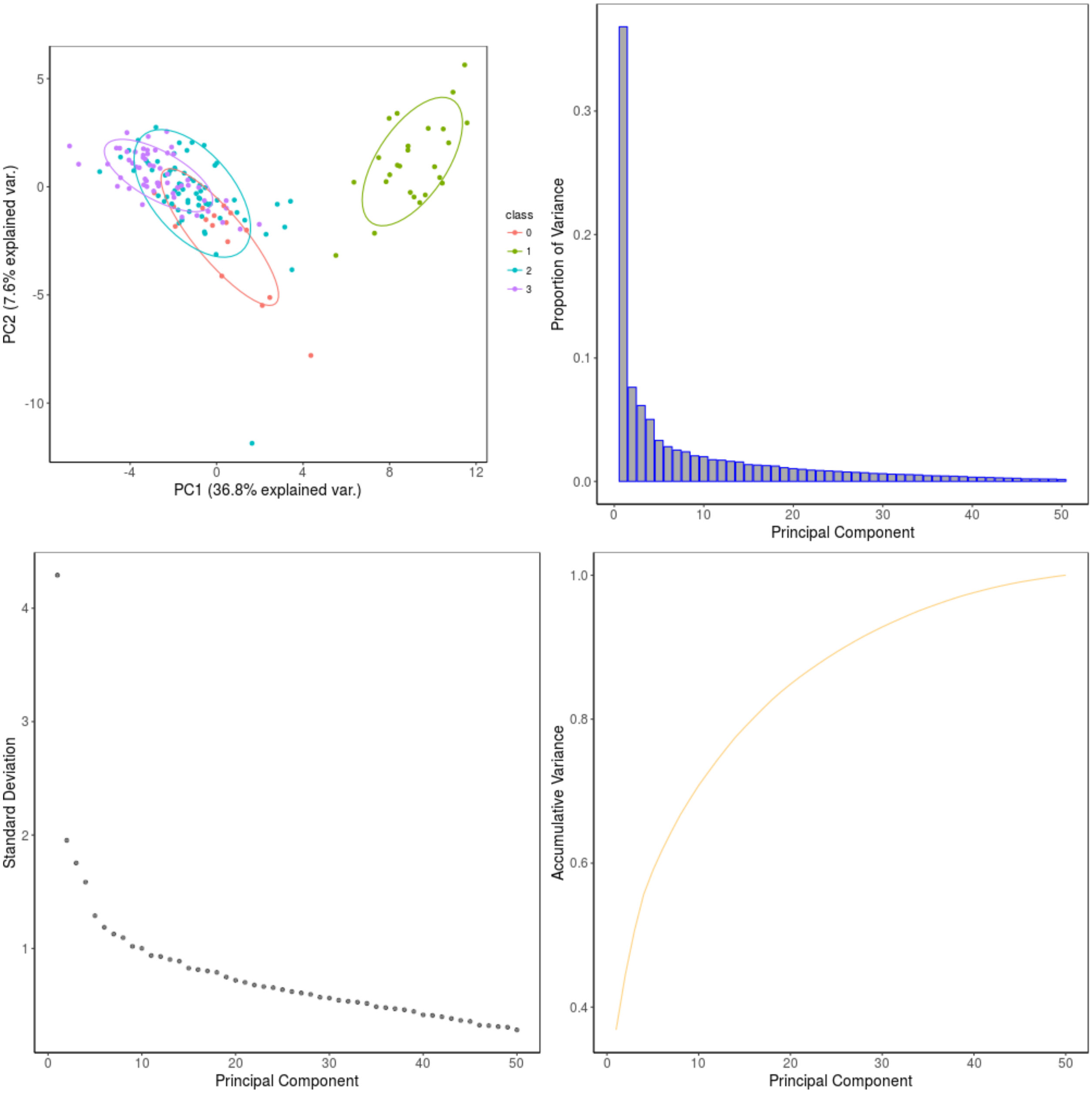

Feature Selection (FS) workflow report

May 18, 2017

Introduction

The report summarizing the Feature Selection pipeline results.

Feature Selection workflow

Univariate canonical correlation (X2) with Principal Component Analysis (PCA) follow by Recursive Feature Elimination (RFE) wrapped with Random Forest (RF).

Dataset

Expression data from normal and prostate tumor tissues (GSE6919_GPL93).

Summary stats from training phase

Table 1: Best model metrics from 10-folds cross-validation resampling.

Variables	Accuracy	Kappa	AccuracySD	KappaSD
1	0.607	0.4154	0.1077	0.153
2	0.642	0.4699	0.1352	0.2115
3	0.6865	0.5459	0.1088	0.1578
4	0.6964	0.5565	0.08473	0.1219
5	0.6774	0.5311	0.07963	0.1125
6	0.7033	0.5683	0.09699	0.1384
7	0.6583	0.5005	0.1111	0.1648
8	0.6585	0.4961	0.0996	0.1501
9	0.6502	0.4843	0.09445	0.1413
10	0.6676	0.5105	0.08141	0.1214
15	0.6783	0.5247	0.07511	0.1153
20	0.6412	0.4735	0.07842	0.115
25	0.6677	0.5093	0.1079	0.158
30	0.6394	0.4637	0.08822	0.1377
35	0.6494	0.4789	0.09674	0.1493
40	0.6577	0.4891	0.08185	0.1298
45	0.6412	0.4587	0.1162	0.196
50	0.6245	0.44	0.08207	0.1332
60	0.6353	0.4543	0.1491	0.2351
70	0.6179	0.4219	0.1063	0.1737
80	0.6345	0.4476	0.1272	0.211
90	0.6005	0.3974	0.1226	0.1939
100	0.5888	0.3764	0.08514	0.1381
165	0.5265	0.2706	0.1244	0.1967

Summary stats from testing phase

Table 2: Classification metrics from twenty class-balanced and randomized runs.

run	Variables	Accuracy	Kappa	AccuracyPValue
1	3	0.7358	0.6161	5.533e-07
2	3	0.7736	0.6733	2.513e-08
3	2	0.7736	0.6603	2.513e-08
4	6	0.7547	0.6391	1.238e-07
5	8	0.717	0.5825	2.255e-06
6	5	0.6226	0.4447	0.0007184
7	4	0.6415	0.487	0.0002658
8	8	0.7925	0.6957	4.606e-09
9	5	0.7736	0.6668	2.513e-08
10	4	0.6981	0.556	8.413e-06
11	5	0.6604	0.5023	9.108e-05
12	4	0.7547	0.6389	1.238e-07
13	9	0.7358	0.6095	5.533e-07
14	5	0.7358	0.6105	5.533e-07
**15**	**6**	**0.8113**	**0.7229**	**7.567e-10**
16	7	0.7358	0.6173	5.533e-07
17	6	0.6981	0.5599	8.413e-06
18	20	0.6226	0.435	0.0007184
19	10	0.6792	0.5253	2.884e-05
20	3	0.717	0.5859	2.255e-06

Accuracy__Mean	Accuracy__SD	Accuracy__Max
0.7217	0.05471	0.8113

Workflow runtime

12.008 minutes

Plots

Visualization of the classification using PCA

- Groups distribution on the first two Principal Components (PC1 and PC2) from the original data (without apply any FS method).

```
## PCA plot not available for this FS workflow setting
```

- Groups distribution on the first two Principal Components (PC1 and PC2) after to apply the FS workflow.

```
## PCA plot not available for this FS workflow setting
```
